# The impact and cost-effectiveness of 9-valent human papillomavirus vaccine in adolescent females in Hong Kong

**DOI:** 10.1186/s12962-021-00328-x

**Published:** 2021-11-20

**Authors:** Tak Hong Cheung, Sally Shuk Yee Cheng, Danny C. Hsu, Queenie Wing-Lei Wong, Andrew Pavelyev, Anuj Walia, Kunal Saxena, Vimalanand S. Prabhu

**Affiliations:** 1 Department of Obstetrics and Gynecology, The Chinese University of Hong Kong, Hong Kong, People’s Republic of China; 2 Global Medical and Scientific Affairs, MSD Asia Ltd, 27/F Lee Gardens Two, 28 Yun Ping Road, Causeway Bay, Hong Kong, People’s Republic of China; 3 MRL, Merck & Co., Inc., 2000 Galloping Hill Road, Kenilworth, NJ 07033 USA; 4 HCL America, Inc., Sunnyvale, CA USA

**Keywords:** Cervical cancer, Cost-effectiveness, Genital warts, Hong Kong, Human papillomavirus, Vaccine

## Abstract

**Introduction:**

In Hong Kong (HK), a single-cohort vaccination program for 10–12-year-old girls with the 9-valent human papillomavirus (HPV) vaccine (9vHPV; types 6/11/16/18/31/33/45/52/58) has been launched. This study assessed the public health impact and cost-effectiveness of implementing routine 9vHPV vaccination (12-year-olds) with or without catch-up 9vHPV vaccination (13–18-year-olds) in HK.

**Methods:**

The health impact and costs of implementing routine 9vHPV vaccination with or without catch-up vaccination over a 100-year time horizon were evaluated using a validated HPV-type transmission dynamic model adapted to the HK population; analyses were performed from a healthcare payer perspective. Routine vaccination (12-year-old girls) and catch-up vaccination (13–18 years) assumed vaccine coverage rates of 70% (base case) and 30%, respectively. The model also assumed herd immunity, lifelong vaccine protection, a discount rate of 3%, and a cost per dose of HK dollars (HKD) 858 [United States dollars (USD) 110] and HKD 1390 (USD 179) for the 2-valent HPV (2vHPV) and 9vHPV vaccines, respectively. HPV disease-related incidence and the incremental cost-effectiveness ratio (ICER) per quality-adjusted-life-year (QALY) were estimated. Cost-effectiveness was determined at a ceiling threshold of HK dollars (HKD) 382,046 (USD 49,142) or 1.0 times the gross domestic product per capita of HK.

**Results:**

Compared with routine 9vHPV alone, routine plus catch-up 9vHPV is projected to reduce cervical cancer incidence by 3.4%. Routine plus catch-up 9vHPV will also reduce genital warts incident cases for males/females by 2.6%/5.4%. The incremental cost-effectiveness ratios were HKD 29,911 (USD 3847)/quality-adjusted life-year (QALY) for routine plus catch-up 9vHPV versus routine 9vHPV alone and HKD 25,524 (USD 3283)/QALY for routine 9vHPV alone versus screening only. Sensitivity analyses indicated that routine plus catch-up 9vHPV compared with routine 9vHPV alone remained cost-effective at coverage rates of 30% and 90%.

**Conclusions:**

This analysis predicts that the current HK vaccination strategy can be considered cost-effective and will provide maximum health benefit. These results support addition of the routine 9vHPV vaccine with or without catch-up 9vHPV vaccination to the regional vaccination program in HK.

**Supplementary Information:**

The online version contains supplementary material available at 10.1186/s12962-021-00328-x.

## Background

Human papillomavirus (HPV) infection causes almost all cases of cervical cancer and is relevant in other cancers, including anal, vulvar, vaginal, penile, and head and neck [1]. These cancers are typically a result of infection with high-risk oncogenic HPV 16/18/31/33/35/39/45/51/52/56/58/66/68/70, whereas nononcogenic HPV types, most frequently HPV 6/11, are associated with genital warts and recurrent respiratory papillomatosis [2].

Cervical cancer is the ninth most common cancer and the ninth leading cause of cancer-related deaths among females in Hong Kong, with crude and age-standardized incidence rates of 12.9 per 100,000 females and 7.6 per 100,000 females, respectively [3]. The overall genital warts incidence rate in Hong Kong is estimated at 203.7 per 100,000 person-years and is higher in males than in females (292.2 vs 124.9 per 100,000 person-years) [4].

Worldwide, the five most prevalent types associated with cervical cancer are HPV 16/18/45/31/33, although geographic variations exist [5]. In Hong Kong, the four most prevalent HPV types associated with cervical cancer are HPV 16/18/52/58 [6]. A study in Hong Kong found HPV 16/52/58 as the primary HPV types attributed to squamous cell carcinoma and cervical intraepithelial neoplasia (CIN)-1/2/3 lesions, indicating that an HPV vaccine that protects against HPV types 52/58 is projected to increase coverage of 11.7% of cervical cancers, 17.7% for CIN-1 lesions, 22.6% for CIN-2 lesions, and 14.4% for CIN-3 lesions, compared with an HPV vaccine that only covers HPV 16/18 [7].

There are currently two vaccines available for the prevention of HPV-related diseases on the market in Hong Kong. The 2-valent HPV (2vHPV) vaccine protects against HPV 16/18, responsible for 70% of cervical cancer cases globally [8], whereas the 9-valent HPV (9vHPV) vaccine offers increased coverage against oncogenic HPV 16/18/31/33/45/52/58, which are associated with approximately 90% of cervical cancers [8], as well as nononcogenic HPV 6/11 associated with 90% of genital warts cases [9].

Both the World Health Organization and the Centre for Health Protection in Hong Kong recommend HPV vaccination along with regular screening for the prevention of cervical cancer [10, 11]. In 2004, a cervical cancer screening program was implemented in Hong Kong that recommends screening by cytology every 3 years following two consecutive normal annual smears in females aged 25–64 years [12]. Although registration with the program increased from 3.6% in 2004 to 20.5% in 2017, cervical Pap testing remained low (57% between 2011 and 2014) [12]. Previous reports have also noted that without a government immunization program, HPV vaccine uptake rates are low in school-aged (11–18 years; approximately 2–9%) and university-aged (< 26 years; 9.7%) females [13–15]. Potential barriers to vaccination include the cost of vaccination and lack of access to information about the HPV vaccine [13, 14].

The Hong Kong government launched a vaccination program with the 9vHPV vaccine during the 2019/2020 school year in all eligible female primary school students in grades 5 and 6. An interim target vaccine coverage of 70% for the 2-dose HPV vaccination schedule was set by the Hong Kong government, with preliminary data reporting a vaccine coverage rate of 85% for the first dose as of December 31, 2020 [16]. The decision to launch this HPV vaccination program was based on a modeling analysis demonstrating that 20 years of routine 9vHPV vaccination in adolescent females in Hong Kong is highly likely to be cost-effective, and the maximum cost for the vaccine to be cost-effective is sensitive to vaccine coverage [17]. These estimates are likely to be conservative as the analysis did not account for other HPV-related diseases (i.e., vulvar, vaginal, or anal cancers and genital warts) or catch-up vaccination [17]. 9vHPV vaccination of school-aged females (10–12-year-olds) has also been shown to be potentially cost-effective in other countries, including Canada and Singapore [18, 19]. A similar model-based analysis conducted for the population of Canada determined that vaccination of 10-year-old girls with the 9vHPV vaccine can be cost-effective, compared with vaccination with the 4-valent HPV (4vHPV) vaccine [18].

Given the high prevalence of HPV 52/58 in Hong Kong, the low historical coverage of HPV vaccination, and plans for the Hong Kong government to introduce a routine HPV vaccination program, there is a need to determine the public health and economic impact of school-based 9vHPV vaccination. We use a model to assess every next cohort at 12 year olds receiving the vaccination with continued coverage of 100 years. Therefore, the aim of this analysis was to assess the impact of a school-based strategy comprising two-dose 9vHPV routine vaccination of 12-year-old girls with or without catch-up 9vHPV vaccination among females aged 13–18 years (two-dose catch-up among those aged 13–14 and three-dose catch-up with 9vHPV vaccination among those aged ≥ 15 years) along with screening in the Hong Kong setting.

## Methods

### Model design

A previously validated HPV-type transmission dynamic mathematical model simulating the natural history of HPV infections and estimating the cost associated with HPV-related diseases [20] was adapted to the Hong Kong setting in order to evaluate the public health and economic impact of HPV vaccination with routine 9vHPV and routine plus catch-up 9vHPV. The current model comprises HPV infection and disease state transitions, lifetime duration of infection-derived immunity, and unvaccinated compartments, which have been previously described in detail [20].

### Target population, study perspective, and time horizon

The target population for routine vaccination was 12-year-old girls, whereas catch-up vaccination was considered for females aged 13–18 years. The health impact was assessed for the entire Hong Kong population across all ages. Costs were assessed using a healthcare payer perspective and impact was measured over a 100-year time horizon because this was considered an appropriate time frame from which the system approached steady state and most benefits and costs of vaccination could be realized. The median age of diagnosis is 49 years for cervical cancer and 62–68 years for other HPV-associated cancers [21]. Given that the target age for routine vaccination in this analysis is 12 years, it may take a long period of time before the benefits of HPV vaccination on costs and outcomes become apparent. Herd immunity was projected over the total population of Hong Kong (7.3 million people) [22] over a 100-year time period in the transmission model.

### Scenarios compared

The vaccination scenarios compared include: (1) screening only, (2) routine 9vHPV vaccination (routine 9vHPV) along with screening, and (3) routine plus catch-up 9vHPV vaccination (routine plus catch-up 9vHPV) along with screening. The focus of this paper will be on routine 9vHPV vaccination with or without catch-up 9vHPV vaccination along with screening.

### Model compartments

The age-structured mathematical model [20] is a single dynamic model that incorporates epidemiology of HPV infection, disease, and economics. Therefore, both the direct and indirect herd immunity benefits and costs of HPV vaccination can be captured by the model for the population over time in a transparent and reproducible manner.

The model structure, equations used, and assumptions have been previously described in detail [20]. At an assumed age of sexual debut of 12 years, individuals enter the population at a sex-specific and sexual-activity-specific rate, moving between successive age groups at an age- and sex-specific rate per year, and exit upon death at an age- and sex-specific per capita death rate per year. An additional age- and stage-dependent death rate is applied for patients with cervical cancer; CIN and genital warts are assumed to carry no additional risk of death. The age-structured model also simulated HPV transmission and the occurrence of CIN, cervical cancer, and genital warts. The acquisition of an infection and progression to disease follow a similar natural history structure, as assumed in previous models for HPV 16/18 [23]. Within the population, patients were divided into epidemiologic categories based on their infection, disease, screening, and treatment status.

The model comprised three connected modules: (1) a demographic variables model describing the age structure of the population (i.e., birth, aging, and death) and behavioral variables describing sexual activity; (2) an epidemiologic model simulating the transmission of HPV 6/11/16/18/31/33/45/52/58, occurrence of genital warts, precancers such as CIN 1/2/3 and vaccine type-related cervical cancer; and (3) a disease variables model describing screening rates, HPV infection, and HPV-related diseases [20].

### Health outcomes and economic outputs

The mathematical model simulated and tracked various outcomes, including numbers of cases of precancers (i.e., CIN-1, CIN-2, CIN-3, vaginal intraepithelial neoplasia 2/3), cervical cancer, vaginal/vulvar cancer, and anal cancer. The model also estimated quality-adjusted life years (QALYs) and total costs, including vaccine-preventable disease costs, and vaccination costs. The incremental cost-effectiveness ratio (ICER) was estimated as the ratio of incremental cost to incremental QALYs. Cost-effectiveness was determined at a ceiling threshold of Hong Kong dollars (HKD) 382,046 [US dollars (USD) 49,142] or 1.0 times the gross domestic product per capita of Hong Kong; this threshold reflected the maximum willingness of decision-makers to pay for an additional QALY [24].

### Inputs

Variables input into the model are shown in Table 1 and Additional file 1: Table S1. Input costs were derived from prices charged to a private case in a public hospital in Hong Kong (Data on file). It is assumed that the private charges in a public hospital setting are equivalent to the cost incurred to the public healthcare provider of the government of Hong Kong.

**Table 1:** Model input parameters

Input parameter			Data source
Demographic variables			
Total population size (males and females), 2016	7,336,600		Hong Kong Census [22]
Sexual behavior variables			
Percentage of the population in the following sexual activity risk groups			
Sexual activity category	Males	Females	Youth Sexuality Study 2011 [27]
Low (0–1 mean sexual partners per year)	80	94	
Medium (2–4 mean sexual partners per year)	15	5	
High (≥ 5 mean sexual partners per year)	5	1	
Mean number of sexual partners per year by the following sexual activity risk groups			
Sexual activity category	Males	Females	Youth Sexuality Study 2011 [27]
Low (0–1 mean sexual partners per year)	0.75	0.92	
Medium (2–4 mean sexual partners per year)	2.52	2.43	
Medium (2–4 mean sexual partners per year)	6.1	7.14	
Screening variables			Hong Kong Department of Health Cervical Screening Programme 2015 [45]
Disease variables			
Cervical cancer			Hong Kong Cancer Registry 2015 [25], Chan 2009 [46], Chan 2011 [7], Lin 2010 [4]
Vaginal/vulvar cancer			Hong Kong Cancer Registry 2015 [25], De Martel 2017 [8]
Anal cancer			Hong Kong Hospital Authority data (Data on file)

HPV vaccination coverage varies by country. Given that this would be a novel program in Hong Kong, we assumed a 70% coverage in 12-year-old girls for routine vaccination for the base case. We assumed 30% coverage in females aged 13–18 years for catch-up vaccination.

The model considered oncogenic HPV 16/18/31/33/45/52/58 for cervical cancer, anal cancer, and CIN 1/2/3, as well as nononcogenic HPV 6/11 for genital warts. For all other HPV-related diseases (i.e., vaginal cancer, vulvar cancer, and vaginal intraepithelial neoplasia 2/3), the model only considered HPV 16/18.

The model also assumed herd immunity, lifelong duration of vaccine protection, a discount rate of 3% for costs and QALYs, and a cost per dose of HKD 858 (USD 110) and HKD 1390 (USD 179) for the 2vHPV and 9vHPV vaccines, respectively. The cost per dose includes the cost of the vaccine plus the administration fee.

### Inputs used for model calibration

Inputs used to calibrate the model include population size (2016 data) [22]; incidence and mortality rates for cervical cancer [25], vaginal/vulvar cancer, 21 genital warts, and anal cancer; annual all-cause mortality rate by sex and age (2016 data) [26]; percentage attribution of cervical cancers of different HPV types; cervical cancer screening rate and age group; sexual behavior variables (including annual mean number of sexual partners by age group; percentage of the population at low, medium, and high sexual activity risk; and mean number of sexual partners per year by sexual activity risk group) [27]; hysterectomy rates by age group [22]; and costs associated with a single episode-of-care for genital warts, cervical cancer screening and visit, colposcopy, biopsy, CIN (1/2/3), cervical cancer, vaginal/vulvar cancer, and anal cancer (Data on file). Additional inputs, such as CIN 1/2/3 treatment variables, were derived from reports of private cases from the Hong Kong Hospital Authority (Data on file).

### Sensitivity analysis

Deterministic one-way sensitivity analyses were conducted to assess the sensitivity of ICER values to variables that have been shown to be impactful to cost effectiveness. One variable was routine vaccination coverage, which was assessed at rates of 30% and 90%. Rationales for selecting different coverage rates: (1) a rate of 30% was chosen, because the HPV catch-up program is more likely to be a public–private partnership with private clinics rather than school-based. As a result, a much lower vaccine uptake rate is expected, and (2) a rate of 90% was based on historical data showing that school-based programs of some vaccines (e.g., DTaP-IPV, MMR, and hepatitis B vaccines) can reach VCR as high as 90% in Hong Kong [28]. Sensitivity analyses also assessed costs (± 10%) and vaccine price discount rates (1% and 5%).

## Results

### HPV-related disease incidence

Compared with routine 9vHPV alone, routine plus catch-up 9vHPV is projected to prevent 769 (3.4% reduction) cases of cervical cancers, over 100 years (Table 2). The model also demonstrated that, compared with routine 9vHPV alone, routine plus catch-up 9vHPV will avert 121 cases of CIN-1 (4.9% reduction), 428 cases of CIN-2/CIN-3 (4.6% reduction), 6401 cases of genital warts among females (5.4% reduction), and 10,090 cases of genital warts among males (2.6% reduction) (Table 2). The number of cases averted by routine plus catch-up 9vHPV and routine 9vHPV alone were both higher, compared with screening only (Table 2).

**Table 2:** Reductions in HPV-related disease incidence for routine plus catch-up 9-valent HPV (9vHPV) female-only vaccination (FOV) vs routine 9vHPV FOV, routine 9vHPV FOV vs screening, and routine plus catch-up 9vHPV FOV vs screening, over 100 years

HPV-related disease	Reduction in cases of HPV-related diseases n(%): Routine plus catch-up^a^ 9vHPV vs routine 9vHPV	Reduction in cases of HPV-related disease n(%): Routine 9vHPV vs screening only	Reduction in cases of HPV-related disease n(%): Routine plus catch-Up^a^ 9vHPV vs screening only
Cervical cancer	769 (3.4)	19,880 (46.9)	20,649 (48.7)
CIN 1	121 (4.9)	3968 (61.8)	4089 (63.7)
CIN 2/3	428 (4.6)	13,766 (59.7)	14,194 (61.5)
Vaginal cancer	2 (3.1)	48 (38.4)	50 (40.2)
Vaginal intraepithelial neoplasia 2/3	2 (3.4)	51 (43.6)	54 (45.5)
Vulvar cancer	5 (3.0)	92 (37.2)	97 (39.1)
Genital warts (female)	6401 (5.4)	306,673 (72.3)	313,074 (73.8)
Genital warts (male)	10,090 (2.6)	534,895 (58.2)	544,985 (59.3)
HPV 6/11-related CIN 1	20 (4.2)	894 (65.3)	914 (66.7)
Anal cancer (female)	55 (2.4)	1087 (32.2)	1142 (33.8)
Anal cancer (male)	42 (1.8)	849 (27.1)	891 (28.4)
Reduction in vaccine-preventable disease costs, % (HKD)	61,366,272 (2.1)	884,053,918 (23.2)	945,420,190 (24.8)

*2vHPV* 2-valent human papillomavirus, *9vHPV* 9-valent human papillomavirus, *CIN* cervical intraepithelial neoplasia *HKD* Hong Kong dollars, *HPV* human papillomavirus

^a^Vaccine uptake was assumed to be 70%, except for the catch-up vaccination scenario, which was assessed at 30% uptake

Compared with routine 9vHPV alone, routine plus catch-up 9vHPV will also result in 3.1%, 3.4%, and 3.0% reductions in the incidences of vaginal cancer, vaginal intraepithelial neoplasia 2/3, and vulvar cancer, respectively (Table 2).

Routine plus catch-up 9vHPV resulted in cumulative reductions in anal cancer incidence of 2.4% and 1.8% for females and males, respectively, compared with routine 9vHPV alone (55 and 42 cases avoided, respectively) (Table 2). Routine plus catch-up 9vHPV and routine 9vHPV alone will lead to a cumulative reduction in anal cancer incidence rates in both females and males (Table 2).

### Economic evaluation and cost-effectiveness

Vaccine-preventable disease costs were reduced by 2.1% [approximately HKD 61.4 million (USD 7.9 million)] for routine plus catch-up 9vHPV versus routine 9vHPV alone (Table 2). Both routine plus catch-up 9vHPV and routine 9vHPV alone would also result in reductions in vaccine-preventable disease costs, compared with screening only (Table 2). Most of the vaccine-preventable disease-related cost reductions associated with routine plus catch-up 9vHPV were attributed to cervical cancer–related treatment costs [HKD 42.5 million (USD 5.5 million) saved]. Long-term trends in estimated healthcare costs avoided over 100 years for routine plus catch-up 9vHPV versus routine 9vHPV alone indicated that most of the cost reductions were associated with HPV 16/18-related treatment costs (59.4% of costs avoided) (Additional file 1: Fig. S1).

Screening-only resulted in the least QALYs, followed by routine 2vHPV alone, routine 9vHPV alone, and routine plus catch-up 9vHPV. ICERs for routine plus catch-up 9vHPV versus routine 9vHPV alone and routine 9vHPV alone versus screening only were HKD 29,911/QALY (USD 3847/QALY) and HKD 25,524/QALY (USD 3283/QALY), respectively (Table 3; Additional file 1: Table S2).

**Table 3:** Cost-effectiveness analysis of routine plus catch-up vs routine 9vHPV female-only vaccination strategies

	Discounted total		Incremental		
	Costs/person (HKD)	QALYs/Person (y)	Costs/person (HKD)	QALYs/person (y)	Costs/QALYs (HKD/y)
Routine 9vHPV FOV	1855.97	27.25912	NA	NA	NA
Routine plus catch-up 9vHPV FOV	1879.45	27.25990	23.48	0.000785	29,911^a^

*9vHPV* 9-valent human papillomavirus, *FOV* female-only vaccination, *HKD* Hong Kong dollar, *ICER* incremental cost-effectiveness ratio, *NA* not applicable, *QALY* quality-adjusted life-year

^a^Incremental costs, QALYs, and ICERS with respect to routine 9vHPV FOV

### Sensitivity analysis

Although cost-effectiveness was sensitive to vaccination coverage, routine vaccination with 9vHPV remained cost-effective at 30% and 90% coverage (ICERs: HKD 17,804/QALY [USD 2290/QALY] and HKD 29,434/QALY [USD 3786/QALY], respectively) compared with routine female-only vaccination with 2vHPV (Additional file 1: Table S3). Furthermore, routine plus catch-up 9vHPV compared with routine 9vHPV alone remained cost-effective at 30%, 70% (base case), and 90% coverage [ICERS: HKD 19,979/QALY (USD 2570/QALY), HKD 29,911/QALY (USD 3847/QALY), and HKD 37,617/QALY (USD 4839/QALY), respectively] (Additional file 1: Table S3). Routine 9vHPV alone compared with 2vHPV also demonstrated cost-effectiveness at 30% and 90% coverage [ICERS: HKD 17,804/QALY (USD 2290/QALY) and HKD 29,434/QALY (USD 3786/QALY), respectively]. Cost-effectiveness was also sensitive to vaccine discount rate for routine plus catch-up 9vHPV versus routine 9vHPV alone, with sensitivity analyses projecting an improvement in the ICER [HKD 5701/QALY (USD 733/QALY)] when the discount rate was decreased to 1%, whereas increasing the discount rate to 5% will increase the ICER to HKD 84,648/QALY (USD 10,888/QALY) (Additional file 1: Table S3). Cost-effectiveness was not sensitive to disease costs for routine plus catch-up 9vHPV versus routine 9vHPV alone, with sensitivity analyses projecting a small decrease in ICER when assuming costs + 10% [HKD 28,764/QALY (USD 3700/QALY)] and a small increase when assuming costs − 10% [HKD 31,043/QALY (USD 3993/QALY)] (Additional file 1: Table S3). Similar trends were observed for routine 9vHPV alone versus routine 2vHPV alone with regards to sensitivity analyses for discount rate (1% and 5%) and costs (± 10%) (Additional file 1: Table S3).

The current analysis evaluated the impact of HPV vaccination with no change in screening patterns. Most models do not change screening patterns over a lifetime, and it is difficult to assess impact on screening rates. Regarding cost figures in Table 3 and Additional file 1: Table S2, the cost per person includes screening, vaccination costs and treatment costs. In addition, these figures are an average cost per person, not just per person vaccinated. Finally, all future costs are discounted, which is why the ICERs are sensitive to discount rate.

## Discussion

We conducted a model-based analysis focusing on routine 9vHPV vaccination of 12-year-old girls (70% coverage) with or without catch-up vaccination (females aged 13–18 years; 30% coverage).

Our findings indicate that 9vHPV routine plus catch-up vaccination of females only yields the most public health benefits (e.g., reductions in HPV-related cervical cancer and genital warts) and may be cost-saving relative to the two alternative options (i.e., screening only and 9vHPV routine vaccination alone) included in this analysis. Sensitivity analyses at assumed vaccination rates of 30% and 90% also demonstrated that the model findings were robust to changes in vaccination coverage. Projections of reduced HPV-related disease incidence and cost-effectiveness with routine 9vHPV vaccination and catch-up compared with screening only in this analysis provides a rationale for implementing a regional vaccination program in 12-year-old females in Hong Kong. Given that the ideal scenario is completion of full-course HPV vaccination prior to sexual debut, the low rates of sexual activity among secondary-school students (age 12–18 years; 1–7%) in Hong Kong [29] further support routine 9vHPV vaccination in 12-year-old females.

The cost-effectiveness of catch-up HPV vaccination programs have been previously evaluated, with most studies demonstrating that catch-up vaccination is cost-effective in females aged 12–18 years [30]. However, results regarding cost-effectiveness of routine HPV plus catch-up vaccination programs were inconsistent from these earlier studies, most likely due to differences in aspects of the models used, such as variation in catch-up strategies and comparator strategies, use of herd immunity, differences in time horizons, country-specific variations (e.g., epidemiology, costs, routine screening practices), and differences in assumptions (e.g., calibration, coverage rates, HPV prevalence, sexual activity patterns) [30]. Choi et al. used an age-structured transmission model adapted to estimate the cost-effectiveness and cost–benefit with routine 9vHPV vaccination in Hong Kong for females aged 12 at vaccine uptake rates of 25%, 50%, and 75% [17]. The study demonstrated cost-effectiveness and cost–benefit compared to screening at rates between 25 and 75% at an estimated upper threshold vaccine cost of USD 444 and USD 689, respectively. However, the analysis only examined benefits of protecting against cervical cancer without accounting for other HPV-related diseases [17]. In contrast, our analysis showed the additional benefits of a catch-up HPV vaccination program.

The current analysis demonstrates that a routine 9vHPV vaccination program for girls aged 10–12 years with or without catch-up 9vHPV vaccination for females aged 13–18 years is cost-effective. Routine plus catch-up 9vHPV vaccination is estimated to provide an additional 2.1% reduction [approximately HKD 61.4 million (USD 7.9 million)] in vaccine-preventable disease costs over routine 9vHPV vaccination alone, resulting in an ICER of HKD 29,911/QALY (USD 3847/QALY). Tay et al. reported similar findings using a validated HPV transmission dynamic model to determine the economic impact of 9vHPV vaccination over the 4vHPV and 2vHPV vaccines in Singapore for females aged 11–12 [19]. The study estimated that 9vHPV vaccination would result in additional overall treatment cost-savings of Singapore dollars (SGD) 61.8 million (USD 46.4 million) and an ICER of SGD 929/QALY (USD 679/QALY), thus highlighting that 9vHPV vaccination is very cost-effective compared with 2vHPV vaccination [19]. The cost-effectiveness demonstrated in our analysis is also consistent with analyses of other HPV vaccination programs, including those analyzed for the 9vHPV vaccine [18, 19, 31–33].

Our estimates of cost-effectiveness of the 9vHPV vaccine are conservative because our model did not account for indirect costs and, in its current form, only models all nine types found in 9vHPV for cervical and anal cancer. For all other cancers, it models only HPV 16/18. A subgroup analysis of 9vHPV vaccination in Asian participants from two international studies (N = 2519) found that HPV 31/33/45/52/58–related disease was prevented with > 90% efficacy (efficacy rates of 90.4%, 94.95%, 100%, and 100% in Japan, Thailand, South Korea, and Hong Kong/Taiwan, respectively) [34]. The higher attribution of HPV 52/58 to HPV burden, and in particular among CIN 1/2/3 lesions, in Hong Kong is more likely to be directly addressed with the 9vHPV vaccine, the only available HPV vaccine that targets these HPV types [7]. The higher attribution of HPV 52/58 to cervical disease is also evident in other Asian countries, with higher prevalence rates reported in Asian (i.e., China, Singapore, Japan, Malaysia, Thailand) versus non-Asian (i.e., USA, UK, Australia) countries for HPV 52/58-associated high-grade cervical lesions (HPV 52, 17.4–28.3% vs 8.9–10.3%; HPV 58, 8.2–16.7% vs 4.9–6.2%) and cervical cancer (HPV 52, 6.2–10.7% vs 0.9–2.4%; HPV 58, 6.2–9.8% vs 1.1–1.6%) [35–43]. Therefore, the external validity of our model may extend beyond Hong Kong to other Asian regions.

## Limitations

A limitation of this study is that it does not account for potential changes to cervical cancer screening with the introduction of routine HPV vaccination. Additionally, there are limited data on the incidence of genital warts in the Hong Kong population because of the variable spectrum of disease manifestation, potential embarrassment for patients, and the transient character of the disease [4]. The model inputs used in this analysis are representative of the cost for all Hong Kong public hospitals, and data gathered are representative of all cases in the Hong Kong population.

The model also assumed no cross-protection against HPV types not targeted by HPV 16/18. Clinical trial results show that the 2vHPV vaccine provided lower and less consistent protection against HPV types 31/33/45 and lasted for a shorter duration than against directly targeted HPV types (HPV 16/18) [44]. Evidence is also lacking in the real-world setting. Therefore, the cross-protection benefit from 2vHPV would be modest in comparison with the 9vHPV vaccine, which directly targets infection against those HPV types. As a result, the assumption is justified.

## Conclusion

This analysis predicts that the current Hong Kong vaccination strategy can be considered cost-effective and will provide maximum health benefit, with greatest reductions in HPV-related disease incidence compared with all other vaccination strategies considered. Considering the low uptake of cervical screening and the high prevalence of HPV types 52/58 in Hong Kong, preventing HPV-related disease represents a concern for the Hong Kong government. These results support the incorporation of a catch-up strategy to the current routine 9vHPV female vaccination program for 12-year-old females in Hong Kong.

## Supplementary Information

**Additional file 1: Table S1.** Model Input Variables Implemented in Transmission Dynamic Model. **Table S2.** Cost-Effectiveness Analysis of the Routine 2vHPV Female-Only Vaccination Strategy vs Screening Only. **Table S3.** Sensitivity Analyses for Cost-Effectiveness of HPV Vaccination Strategies. **Figure S1.** Estimated healthcare costs avoided over 100 years by HPV type with routine plus catch-up FOV with the 9vHPV vaccine versus (A) FOV with the 2vHPV vaccine and (B) FOV with the 9vHPV vaccine.

## Data Availability

All relevant data are contained within the article. The datasets used and/or analyzed in the current study are available from the corresponding author on reasonable request.

## Abbreviations

2vHPV: 2-Valent HPV
9vHPV: 9-Valent human papillomavirus (HPV) vaccine
CIN: Cervical intraepithelial neoplasia
HK: Hong Kong
HKD: Hong Kong dollar
HPV: Human papillomavirus
ICER: Incremental cost-effectiveness ratio
QALY: Quality-adjusted life-year
USD: US dollars
