# Supplementary material for: The impact and cost-effectiveness of 9-valent human papillomavirus vaccine in adolescent females in Hong Kong
Source: Cost Eff Resour Alloc. 2021 Nov 20;19:75. doi: 10.1186/s12962-021-00328-x (PMC8606050; doi:10.1186/s12962-021-00328-x)

**Additional file 1**

## **Table S1**. Model Input Variables Implemented in Transmission Dynamic Model

| **Demographic variables** | | |
| --- | --- | --- |
| Annual all-cause mortality rate by gender and age (%). The rate, which represents the fraction of individuals in the age and gender group who are expected to die over the course of 1 year, should be between 0 and 1^(25)^ | Males | Females |
| 0–<1 year | 0.0552 | 0.0505 |
| 1–10 years | 0.0079 | 0.0057 |
| 11–14 years | 0.0083 | 0.0079 |
| 15–19 years | 0.0215 | 0.0164 |
| 20–24 years | 0.0322 | 0.0169 |
| 25–29 years | 0.0466 | 0.0188 |
| 30–34 years | 0.0622 | 0.0256 |
| 35–39 years | 0.0833 | 0.0382 |
| 40–44 years | 0.1253 | 0.0749 |
| 45–49 years | 0.1990 | 0.1050 |
| 50–54 years | 0.3107 | 0.1736 |
| 55–59 years | 0.5293 | 0.2555 |
| 60–64 years | 0.8374 | 0.3985 |
| 65–69 years | 1.2641 | 0.5879 |
| 70–74 years | 2.1524 | 0.9650 |
| 74–79 years | 3.4555 | 1.7129 |
| 80–84 years | 6.0256 | 3.3423 |
| 85+ years | 12.2915 | 8.9195 |
| **Sexual behavior variables** | | |
| **Data for age groups other than those aged 15–19 years are not available in Hong Kong** | | |
| Population annual mean number of sexual partners by sex and age group^(26)^ | Males | Females |
| 13–14 years | 0.06 | 0.06 |
| 15–19 years | 1 | 1.78 |
| 20-29 years | 1.04 | 1.04 |
| 25-29 years | 1.04 | 1.04 |
| 30–34 years | 0.92 | 0.92 |
| 35–39 years | 0.92 | 0.92 |
| 40–44 years | 0.92 | 0.92 |
| 45-49 years | 0.92 | 0.92 |
| 50-54 years | 0.58 | 0.58 |
| 55+ years | 0.58 | 0.58 |
| **Screening variables^(12, 43)^** | | |
|  | Female Only | |
| Percentage of female population that receives cervical cancer screening test at least once every 3 years | 13.04% | |
| Cervical cancer screening rate by age group, per year, % |  | |
| 10–14 years | 0.00% | |
| 15–19 years | 0.00% | |
| 20–24 years | 0.00% | |
| 25–29 years | 2.34% | |
| 30–34 years | 4.17% | |
| 35–39 years | 4.59% | |
| 40–44 years | 4.87% | |
| 45–49 years | 4.81% | |
| 50–54 years | 4.86% | |
| 55–59 years | 4.75% | |
| 60–64 years | 3.83% | |
| ≥65 years | 0.45% | |
| Percentage of females with a follow-up screening test following an abnormal Pap test result | 82.5% | |
| **Disease variables** | | |
| **Data only available in Hong Kong are age-specific incidence rate, mortality rate, and stage distribution of cervical cancer. The stage-specific mortality rate is not available** | | |
| Age and stage-specific cervical cancer mortality rates per year. The rate, which represents the fraction of individuals in the age group with cancer at the given stage who are expected to die over the course of 1 year, should be between 0 and 1 | Females Only | |
| LCC |  | |
| 15–29 years | 0 | |
| 30–39 years | 0.0163 | |
| 40–49 years | 0.0807 | |
| 50–59 years | 0.1108 | |
| 60–69 years | 0.064 | |
| 70+ years | 0.31 | |
| RCC |  | |
| 15–29 | 0.2685 | |
| 30–39 | 0.15447 | |
| 40–49 | 0.1097 | |
| 50–59 | 0.1496 | |
| 60–69 | 0.1728 | |
| 70+ | 0.3671 | |
| DCC |  | |
| 15–29 | 0.5335 | |
| 30–39 | 0.75 | |
| 40–49 | 0.7196 | |
| 50–59 | 0.6407 | |
| 60–69 | 0.3985 | |
| 70+ | 0.398 | |
| Population level hysterectomy rates by age group, per year, % (Data on file)^(21)^ | Female Only | |
| 15–24 years | 0.0005% | |
| 25-29 years | 0.0037% | |
| 30–34 years | 0.0128% | |
| 35–39 years | 0.0335% | |
| 40–44 years | 0.1081% | |
| 45–54 years | 0.1828% | |
| 55 years + | 0.0981% | |
| **Economic variables** | | |
| Costs of an episode-of-care derived from private cases in public hospitals in Hong Kong (Data on file) | | |
| Genital warts: female | HKD 1110 | |
| Genital warts: male | HKD 1110 | |
| Cervical cancer |  | |
| Cervical cancer screening and visit | HKD 1210.00 | |
| Colposcopy | HKD 4080.00 | |
| Biopsy | HKD 4580.00 | |
| CIN-1 episode-of-care | HKD 5790.00 | |
| CIN-2 episode-of-care | HKD 18,790.00 | |
| CIN-3 episode of care | HKD 18,790.00 | |
| LCC | HKD 225,790.00 | |
| RCC | HKD 267,790.00 | |
| DCC | HKD 80,000.00 | |
| Vaginal cancer |  | |
| Consultation | HKD 1210.00 | |
| Diagnostic screening | HKD 4080.00 | |
| Biopsy | HKD 4580.00 | |
| Stage I/IIA local cancer | HKD 162,945.00 | |
| Stage IIB/IIIB regional cancer | HKD 169,870.00 | |
| Stage IV distant cancer | HKD 50,000.00 | |
| Vulvar cancer |  | |
| Stage I/IIA local cancer | HKD 131,340.00 | |
| Stage IIB/IIIB regional cancer | HKD 193,030.00 | |
| Stage IV distant cancer | HKD 50,000.00 | |
| Anal cancer | Female and male | |
| Stage I/IIA local cancer | HKD 245,260.00 | |
| Stage IIB/IIIB regional cancer | HKD 258,235.00 | |
| Stage IV distant cancer | HKD 12,000.00 | |
| **Treatment variables (Data on file)** | | |
| Proportion of CIN/CIS is treated, by stage | Female only | |
| CIN-1 | 3.8% | |
| CIN-2 | 95.5% | |
| CIN-3 | 95.5% | |
| CIS | 95.5% | |
| Proportion of treated genital warts cases | 75% (assumed for both male and female) | |

CIN, cervical intraepithelial neoplasia; CIS, carcinoma in situ; DCC, distant cervical cancer; HKD, Hong Kong dollars; LCC, local cervical cancer; RCC, regional cervical cancer.

## **Table S2**. Cost-Effectiveness Analysis of the Routine 2vHPV Female-Only Vaccination Strategy vs Screening Only

|  | **Discounted Total** | | **Incremental** | | |
| --- | --- | --- | --- | --- | --- |
|  | **Costs/Person (HKD)** | **QALYs/Person (y)** | **Costs/Person (HKD)** | **QALYs/Person (y)** | **Costs/QALYs (HKD/y)** |
|  | | | | | |
| Screening Only | 1613.18 | 27.24960 | NA | NA | NA |
| Routine 2vHPV FOV | 1766.93 | 27.25560 | 153.75 | 0.0059946 | 25,651^a^ |

^a^Incremental costs, QALYs, and ICERS with respect to screening-only scenario.

*2vHPV* 2-valent human papillomavirus, *FOV* female-only vaccination, *HKD* Hong Kong dollar, *ICER* incremental cost-effectiveness ratio, *NA* not applicable, *QALY* quality-adjusted life-year.

## **Table S3**. Sensitivity Analyses for Cost-Effectiveness of HPV Vaccination Strategies

| Scenario | ICER 9vHPV vs 2vHPV | ICER 9vHPV with Catch-Up vs 9vHPV |
| --- | --- | --- |
| **VCR** |  |  |
| 70% coverage for routine vaccination (base case) | 25,308 | 29,911 |
| 30% coverage for routine vaccination | 17,804 | 19,979 |
| 90% coverage for routine vaccination | 29,434 | 37,617 |
| **Discount rate** |  |  |
| 1% discount | 10,217 | 5,701 |
| 5% discount | 51,683 | 84,648 |
| **Costs** |  |  |
| +10% | 23,810 | 28,764 |
| –10% | 26,806 | 31,043 |

2vHPV, 2-valent human papillomavirus; 9vHPV, 9-valent human papillomavirus; HPV, human papillomavirus; ICER, incremental cost-effectiveness ratio.

**Figure S1**. Estimated healthcare costs avoided over 100 years by HPV type with routine plus catch-up FOV with the 9vHPV vaccine versus (A) FOV with the 2vHPV vaccine and (B) FOV with the 9vHPV vaccine. 2vHPV, 2-valent human papillomavirus; 9vHPV, 9-valent human papillomavirus; FOV, female-only vaccination; HKD, Hong Kong dollars; HPV, human papillomavirus; ICER, incremental cost-effectiveness ratio.


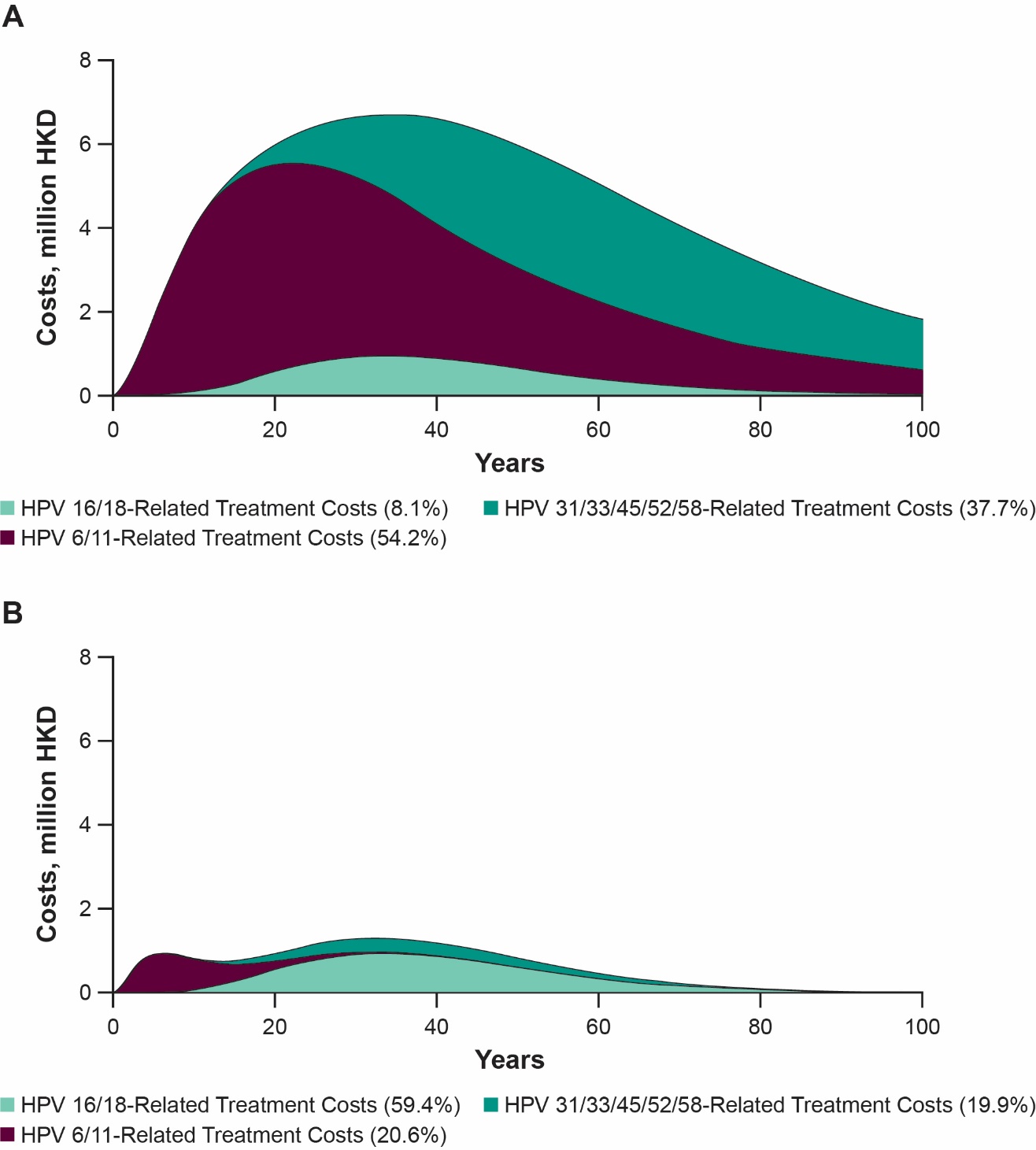

Supplement: Supplementary file 1 — Additional file 1: Table S1. Model Input Variables Implemented in Transmission Dynamic Model. Table S2. Cost-Effectiveness Analysis of the Routine 2vHPV Female-Only Vaccination Strategy vs Screening Only. Table S3. Sensitivity Analyses for Cost-Effectiveness of HPV Vaccination Strategies. Figure S1. Estimated healthcare costs avoided over 100 years by HPV type with routine plus catch-up FOV with the 9vHPV vaccine versus (A) FOV with the 2vHPV vaccine and (B) FOV with the 9vHPV vaccine. [file 12962_2021_328_MOESM1_ESM.docx]
